# Immunoproteomic analysis of the serum IgG response to cell wall-associated proteins of *Staphylococcus aureus* strains belonging to CC97 and CC151

**DOI:** 10.1186/s13567-023-01212-7

**Published:** 2023-09-18

**Authors:** Shauna D. Drumm, Paul Cormican, Rebecca A. Owens, Jennifer Mitchell, Orla M. Keane

**Affiliations:** 1 Animal and Bioscience Department, Teagasc, Grange, Dunsany, Co. Meath, Ireland; 2 School of Biomolecular and Biomedical Science, University College Dublin, Belfield, Dublin 4, Ireland; 3 Department of Biology, Maynooth University, Maynooth, Co. Kildare Ireland; 4 Seed Testing Laboratory, DAFM Laboratories, Backweston, Celbridge, Co. Kildare Ireland

**Keywords:** *Staphylococcus aureus*, bovine mastitis, cell wall-anchored proteins, immunogenicity

## Abstract

CC97 and CC151 are two of the most common *Staphylococcus aureus* lineages associated with bovine intramammary infection. The genotype of the infecting *S. aureus* strain influences virulence and the progression of intramammary disease. Strains from CC97 and CC151 encode a distinct array of virulence factors*.* Identification of proteins elaborated in vivo will provide insights into the molecular mechanism of pathogenesis of these lineages, as well as facilitating the development of tailored treatments and pan-lineage vaccines and diagnostics. The repertoire of genes encoding cell wall-anchored (CWA) proteins was identified for *S. aureus* strains MOK023 (CC97) and MOK124 (CC151); MOK023 encoded more CWA proteins than MOK124. Serum collected during an in vivo challenge trial was used to investigate whether the humoral response to cell wall proteins was strain-specific. Immunoproteomic analysis demonstrated that the humoral response in MOK023-infected cows predominantly targeted high molecular weight proteins while the response in MOK124-infected cows targeted medium or low molecular weight proteins. Antigenic proteins were identified by two-dimensional serum blotting followed by mass spectometry-based identification of immunoreactive spots, with putative antigens subsequently validated. The CWA proteins ClfB, SdrE/Bbp and IsdA were identified as immunogenic regardless of the infecting strain. In addition, a number of putative strain-specific imunogens were identified. The variation in antigens produced by different strains may indicate that these strains have different strategies for exploiting the intramammary niche. Such variation should be considered when developing novel control strategies including vaccines, therapeutics and diagnostics.

**Supplementary Information:**

The online version contains supplementary material available at 10.1186/s13567-023-01212-7.

## Introduction

*Staphylococcus aureus* is a major pathogen associated with bovine intramammary infection (IMI) and is responsible for substantial economic losses in the dairy industry [1, 2]. A wide variety of *S. aureus* sequence types have been associated with IMI, with clonal complex (CC) 97 and CC151 being two of the most common, globally-distributed, bovine-adapted lineages [3]. The *S. aureus* genome consists of a core genome, found in all strains of the species, and a variable genome, found in only some strains, with many virulence genes encoded within the variable genome. Strains within a lineage generally have a similar variable genome but this differs from the variable genome of other lineages as the *S. aureus* restriction-modification system limits horizontal gene transfer between lineages [4]. Significant differences in the virulence gene content of CC97 and CC151 strains have been demonstrated. CC151 strains encode a large number of toxins but few genes encoding cell wall-anchored (CWA) proteins involved in host cell adherence, internalisation and biofilm formation. In contrast, CC97 strains encode few toxins but have an array of genes encoding CWA proteins [5–7].

A variety of in vitro studies have demonstrated significant differences between isolates from CC97 and CC151 in a selection of virulence traits, including host cell adherence and internalisation, biofilm production and elicitation of a host immune response [8–11]. In vivo studies in which cows were challenged with strain Newbould 305 (CC97) or RF122 (CC151), demonstrated that Newbould 305 generally causes mild or sub-clinical mastitis while RF122 gives rise to severe clinical mastitis [12–15]. Indeed, a recent in vivo study directly compared the host response to *S. aureus* strains MOK023 (CC97) and MOK124 (CC151). Intramammary challenge with MOK124 resulted in more overt clinical signs, higher somatic cell count, higher milk IL-1β, IL-8 and anti-*S. aureus* IgG and a greater drop in milk yield compared to challenge with MOK023 [16]. Milk somatic cell transcriptomic analysis demonstrated that each strain induced a characteristic host response [17]. This suggests that these strains may have developed differing strategies for the exploitation of the intramammary niche and may differ in their mode of pathogenicity.

Understanding the impact of strain variation on disease epidemiology, clinical presentation and the host response to bovine IMI is crucial for the development of novel prevention and control strategies. In particular, the development of novel vaccines and/or diagnostic tools would limit the negative impact of this disease on animal health and welfare and enable a reduction in the use of antimicrobials in the dairy industry. Currently, much *S. aureus* research has focussed on the potential of CWA proteins as protective antigens [18–21]. These proteins are common vaccine targets due to their location on the surface of the bacterial cell and their role in host cell interaction, nutrient utilisation and immune evasion. However, human *S. aureus* vaccines based on CWA proteins have to-date failed to provide protection [22]. One recently elaborated explanation for this is that prior exposure to *S. aureus* may induce an “immune imprint” with subsequent vaccination resulting in the preferential recall of non-protective antibodies. These antibodies can compete with protective antibodies, reducing opsonophagocytosis and further impairing a protective response [23]. Despite this caveat, the identification of immunogenic proteins has a number of advantages; it enables epitope mapping to identify protective protein domains as well as enabling the identification of proteins and virulence factors expressed in vivo, which may be exploitable as therapeutic targets or diagnostic markers. However, *S. aureus* IMI control strategies must account for the extensive genomic diversity between bovine-adapted strains and lineages. For example, strains belonging to CC151 lack many genes encoding CWA proteins [5, 6] while ST71 strains do not encode the *ica* operon [5], the protein products of which are responsible for poly-N-acetylglucosamine biosynthesis [24], a major target of one of the few licenced vaccines [25].

In this study a genomic and immunoproteomic approach was used to identify the repertoire of, and interrogate the humoral immune responses to, CWA proteins expressed by CC97 and CC151 strains of *S. aureus* during bovine IMI. Key cell wall proteins expressed by each strain that elicited an IgG response in infected cows were identified and validated. Comparison of the immunogenic molecules elaborated in vivo by each strain facilitates the identification of pan-strain and strain-specific antigens.

## Materials and methods

### *S. aureus* strains and culture conditions

*S. aureus* strains MOK023 and MOK124 were recovered from milk of cows presenting with mastitis as described previously [26]. Strain-typing demonstrated they belong to ST3170 (CC97) and ST151 (CC151) respectively [5]. *S. aureus* strains were preserved in Trypticase Soy Broth (TSB) (LabM, Heywood, UK) supplemented with 15% (v/v) glycerol at −80 °C. When required, strains were recovered on Trypticase Soy Agar (TSA) (LabM) at 37 °C overnight. Strains were single colony purified every 7 days onto fresh TSA. For liquid cultures, strains were grown in Trypticase Soy Broth (TSB) at 37 °C and 200 rpm.

### Genome assembly and annotation

Whole genome sequence data for MOK023 and MOK124 are available [5, 16], accession numbers SRS775827 and SRS2841713 respectively. Quality control checks were carried out on both forward and reverse reads using the FastQC software [27]. Draft genomes were assembled and annotated as previously described [28]. For each putative protein, subcellular location was predicted using PSORTb v3.0 [29].

### Identification of putative CWA proteins

In *S. aureus* 25 CWA proteins have been described [30]. Two cell wall-anchoring domains exist, each the target of one of two proteolytic enzymes, Sortase A or Sortase B, responsible for anchoring the protein in the cell wall. Substrates of Sortase A have a tripartite motif, including an LPxTG cleavage motif, at the C-terminal while substrates of Sortase B have a NPQTN cleavage motif [31]. Predicted proteins containing either of the sortase cleavage motifs were identified. Subsequently, a hidden markov model, designed to directly predict Gram positive bacterial CWA proteins based on the presence of the tripartite motif [32], was used to predict if proteins carrying a sortase cleavage motif were putative CWA proteins. BLASTP was used for the identification of putative CWA proteins and determining the presence of sequence or structural variation. Structural variation in *clfA*, *isdH* and *sasA* for MOK124 and *sasC* for MOK023 was confirmed by Sanger sequencing.

### Preparation of *S. aureus* cell wall-associated proteins

*S. aureus* strains were grown overnight in 5 mL of TSB at 37 °C with 200 rpm orbital shaking. The following morning, the overnight cultures were diluted 1:100 into 250 mL of fresh TSB and incubated at 37 °C, 200 rpm until mid-exponential phase (OD600nm ~0.4). Cultures were then centrifuged at 1000 × *g* for 30 min and the supernatant removed. Pelleted cells were resuspended in phosphate buffered saline (PBS), adjusted to an OD600nm of 10 and centrifuged at 5000 × *g* for 2 min. The supernatant was removed and the cell pellets resuspended in Digestion Buffer (30% raffinose in 20 mM MgCl_2_, 50 mM Tris–HCl, pH7.5) containing 1 X EDTA-free protease inhibitor cocktail (Sigma Aldrich, St Louis, USA). Lysostaphin from *Staphylococcus staphylolyticus* (Sigma Aldrich) was added to a final concentration of 0.2 mg/mL before incubation at 37 °C for 30 min. Cell wall-associated proteins were harvested by supernatant collection after centrifugation at 5000 × *g* for 15 min. Nucleic acids were removed by adding 100 X Protease Inhibitor Mix (GE Healthcare, Chicago, USA) and 100 X Nuclease Mix (GE Healthcare) to a final concentration of 1 X and allowing digestion for 50 min at room temperature with frequent mixing. Desalting was carried out using the PD-10 Desalting Column (GE Healthcare), following the gravity flow protocol, as per the manufacturer’s instructions. The elution step was repeated twice. For protein concentration and buffer exchange, 15 mL of prepared cell wall-associated proteins was added to a 3 kDa molecular weight cutoff filter (Amicon, Millipore, Darmstadt, Germany) and centrifuged at 4000 × *g* until 1 mL of supernatant remained in the unit. The flow through was discarded, and PBS added to bring sample back to 15 mL. The filter device was again centrifuged at 4000 × *g* until 1 mL of supernatant remained in the unit and flow through discarded. The PBS wash was repeated once and Isoelectric Focusing (IEF) Rehydration buffer (10 mM Tris, 8 M Urea, 2 M Thiourea, 4% (w/v) CHAPS, 1% (v/v) TritonX-100) or Storage buffer (6 M Urea, 2 M Thiourea, 0.1 M Tris–HCl pH 8.6, filter sterilized) was added to bring the sample back to 15 mL. The filter device was centrifuged at 4000 × *g* until no more flow through passed and the concentrated supernatant removed to a fresh 1.5 mL Eppendorf tube. Protein quantification was performed using the 2-D Quant kit (GE Healthcare), as per the manufacturer’s instructions.

### Gel electrophoresis

Protein samples were separated by either one-dimensional (1D) or two-dimensional (2D) SDS-PAGE, as previously described [28]. Briefly, protein samples were prepared for 1D electrophoresis by adding LDS Sample Buffer (NuPAGE, Thermo Scientific, Waltham, USA) to a final concentration of 1 X, boiled for 10 min and separated on 8–12% SDS-PAGE gels using the Mini-PROTEAN Tetra Cell (Bio-Rad, Hercules, USA). Following electrophoresis, proteins were either stained by coomassie dye or immobilised onto PVDF membrane at 20 V for 1.5 h using the Bio-Rad Trans-Blot SD Semi-Dry Electrophoretic Transfer Cell. For 2D electrophoresis, protein samples were adjusted to a concentration of 60 µg in 125 µL of IEF Rehydration Buffer containing 0.8% (w/v) IPG pH 3–10 NL buffer (GE Healthcare), 15 mg/mL DeStreak Reagent (GE Healthcare) and a trace amount of bromophenol. Proteins were separated according to their isoelectric point (p*I*) using non-linear gradient strips (Immobiline Drystrip pH 3–10 NL; GE Healthcare) on the Agilent (Santa Clara, USA) 3100 OFFGEL Fractionator until a kVh of 8 was reached. Strips were then reduced for 10 min with agitation in Equilibration buffer (30% (v/v) glycerol, 2% (w/v) SDS, 6 M urea, 50 mM Tris–HCL pH 8.8) containing 2% (w/v) dithiothreitol (DTT) followed by alkylation for 10 min with agitation in Equilibration buffer containing 2.5% (w/v) iodoacetamide (IAA). Proteins were then electrophoresed on 10% SDS-PAGE gels using the Mini-PROTEAN Tetra cell (Bio-Rad). Following electrophoresis, gels were either stained by silver nitrate rapid staining, as described by [33], or immobilised onto PVDF membrane, as described above for 1D SDS-PAGE.

### Serum blotting

Bovine sera came from a *S. aureus* intramammary infection trial previously described [16] and consisted of sera from 5 and 4 cows infected with MOK023 and MOK124 respectively. Serum samples were from 5 time-points over the course of infection: pre-infection (day 0) and 7, 14, 21 and 29 days post-infection. Serum from day 29 post-infection was missing for one cow (582) from the MOK124 group.

Membranes were blocked in 10% non-fat, dried milk in PBS for 1 h at room temperature followed by 3 × 10 min washes in PBS. Membranes were then incubated overnight at 4 °C with bovine sera diluted 1:2000 in PBS. Subsequently, membranes were washed in PBS as described above, before incubating in the dark for 2 h at room temperature with horseradish peroxidase (HRP)-conjugated goat anti-cow IgG (Abcam, Cambridge, UK), diluted 1:5000 in filter-sterilised 5% BSA in PBS. The membranes were subsequently washed 4 × 10 min in PBS. The membranes were incubated with SuperSignal West Pico PLUS Chemiluminescent Substrate (Thermo Scientific) and imaged using the Omega Lum C Imaging System (Aplegen, Pleasanton, USA).

### Identification of immunogenic proteins

Serum blotted 2D membranes were manually aligned to the corresponding stained 2D gel. Spots identified as immunogenic were excised from the gel and each spot placed in a separate sterile eppendorf tube. Excised spots were prepared for mass spectrometry, with overnight digestion at 37 °C, as described by [34]. Following digestion, the supernatant was harvested and peptides dried in a new sterile tube.

### Mass spectrometry analysis

Dried digested samples were resuspended in 0.5% (v/v) TFA before desalting using Pierce™ C18 spin tips (Thermo Scientific). Desalted peptides were resuspended in Loading Solution (0.05% (v/v) TFA, 2% (v/v) acetonitrile) and analysed on a Q Exactive™ Hybrid Quadrupole-Orbitrap™ Mass Spectrometer coupled to an UltiMate™ 3000 RSLCnano System (Thermo Scientific), according to the method of Owens et al. [35]. Mass spectrometry data were analysed using Proteome Discoverer software (v1.4; Thermo Scientific™), against protein databases created for each strain, using the SEQUEST algorithm with the following settings (i) trypsin was selected as the cleavage enzyme with up to 2 missed cleavages allowed (ii) oxidation of methionine was set as a variable modification and (iii) carbamidomethylation of cysteine was set as a fixed modification. Results were filtered using the Percolator module and only medium confidence peptides (False Discovery Rate (FDR) < 0.05) were retained. Further filtering was performed to remove proteins identified by only a single unique peptide. Where more than one protein was detected in a given excised spot, candidate proteins were identified based on (i) predicted location in the cell wall, (ii) predicted molecular weight, (iii) predicted p*I* and (iv) the number of unique peptides.

### Recombinant protein expression and blotting

The plasmids used for recombinant protein expression are listed in Table 1. Plasmids encoding ClfA, ClfB and FnbpB were provided by Prof. Joan Geoghegan, Trinity College Dublin. Plasmids encoding IsdA and SirA were provided by Prof. Michael Murphy, The University of British Columbia while the plasmid encoding SdrD was provided by Prof. Mona Johannessen, The Arctic University of Norway. All plasmids were transformed into BL21 (DE3) competent *Escherichia coli* (New England BioLabs, Ipswich, USA) and the integrity of the plasmid insert confirmed by Sanger sequencing. Transformed *E. coli* strains were stored in Luria–Bertani (LB) Broth (Sigma Aldrich) supplemented with 15% (v/v) glycerol at −80 °C until required. Strains were recovered on LB (Sigma Aldrich) plates, containing 100 µg/mL ampicillin or 30 µg/mL kanamycin as required, at 37 °C overnight. For liquid cultures, strains were grown in LB broth, containing appropriate antibiotics as required, at 37 °C with 250 rpm orbital shaking.

**Table 1:** **Plasmids used in this study.**

Protein of interest	Plasmid description	*S. aureus* strain used for gene cloning	Protein domain or region cloned	Construct name	Antibiotic resistance marker	Final IPTG Concentration	References
ClfA	pQE30:ClfA N1N2N3	Newman	Complete A domain minus signal sequence AA 40-559	pCF40	Ampicillin	100 µM	[61]
ClfB	pQE30:ClfB N1N2N3	DU5966	Complete A domain minus signal sequence AA 44-542	pN123	Ampicillin	100 µM	[62]
FnbpB	pQE30:FnBPB N1N2N3	8325-4	Complete A domain minus signal sequence AA 37-480	pQE30::rFnBPB37–480	Ampicillin	100 µM	[63]
IsdA	pGEX2T TEV:IsdA	N315	Complete protein minus signal sequence and anchoring domain AA 48-316	GST-IsdA	Ampicillin	300 µM	[64]
SdrD	pSdrD-pRSETB-N-His	8325-4	Complete protein minus signal sequence and anchoring domain AA 53-1315	pRSETB-SdrD	Ampicillin	40 µM	[65]
SirA	pET28a:SirA_T37_	N315	Complete protein minus signal sequence AA 37-330	SirA_T37_	Kanamycin	300 µM	[66]

For recombinant protein expression*, E. coli* strains were inoculated into 5 ml of LB broth, with appropriate antibiotics, and grown at 37 °C, 250 rpm for 4 h. Recombinant protein production was induced for 3 h with IPTG (final concentration given in Table 1). Following induction, cultures were centrifuged at 1000 × *g* for 30 min and the supernatant removed. Cell pellets were resuspended in PBS and adjusted to the same OD600nm. Cells were then centrifuged at 3500 × *g* for 3 min, resuspended in 1 X LDS buffer and boiled for 10 min. The whole cell lysates were separated by 1D SDS-PAGE, using 8–12% gels. Gels were stained with coomassie dye or proteins immobilised onto PVDF membrane. Blots were probed with bovine sera as described above. A lysate of untransformed *E. coli* and transformed, uninduced *E. coli* were included as controls.

## Results

### Identification of genes encoding putative CWA proteins of *S. aureus* MOK023 and MOK124

MOK023 and MOK124 were predicted to encode 19 and 10 intact *S. aureus* CWA proteins, respectively (Additional file 1). Neither strain encoded *cna*, *bap*, *sasL*, *pls* or *sasX* while *fnbB*, *sasG*, *sasK* and *sdrD* were also missing from the genome of MOK124. MOK023 encoded *sdrE* while MOK124 encoded *bbp*, the allelic variant of *sdrE*. Structural variation, predicted to prevent anchoring of the cognate protein in the cell wall, was identified in *sasC* for MOK023 and *clfA*, *isdH*, *sasA*, *sasC*, *sdrC* and *spa* for MOK124 (Additional file 1).

### Characterisation of the humoral response to *S. aureus* cell wall-associated proteins from MOK023 and MOK124

**Figure 1:**
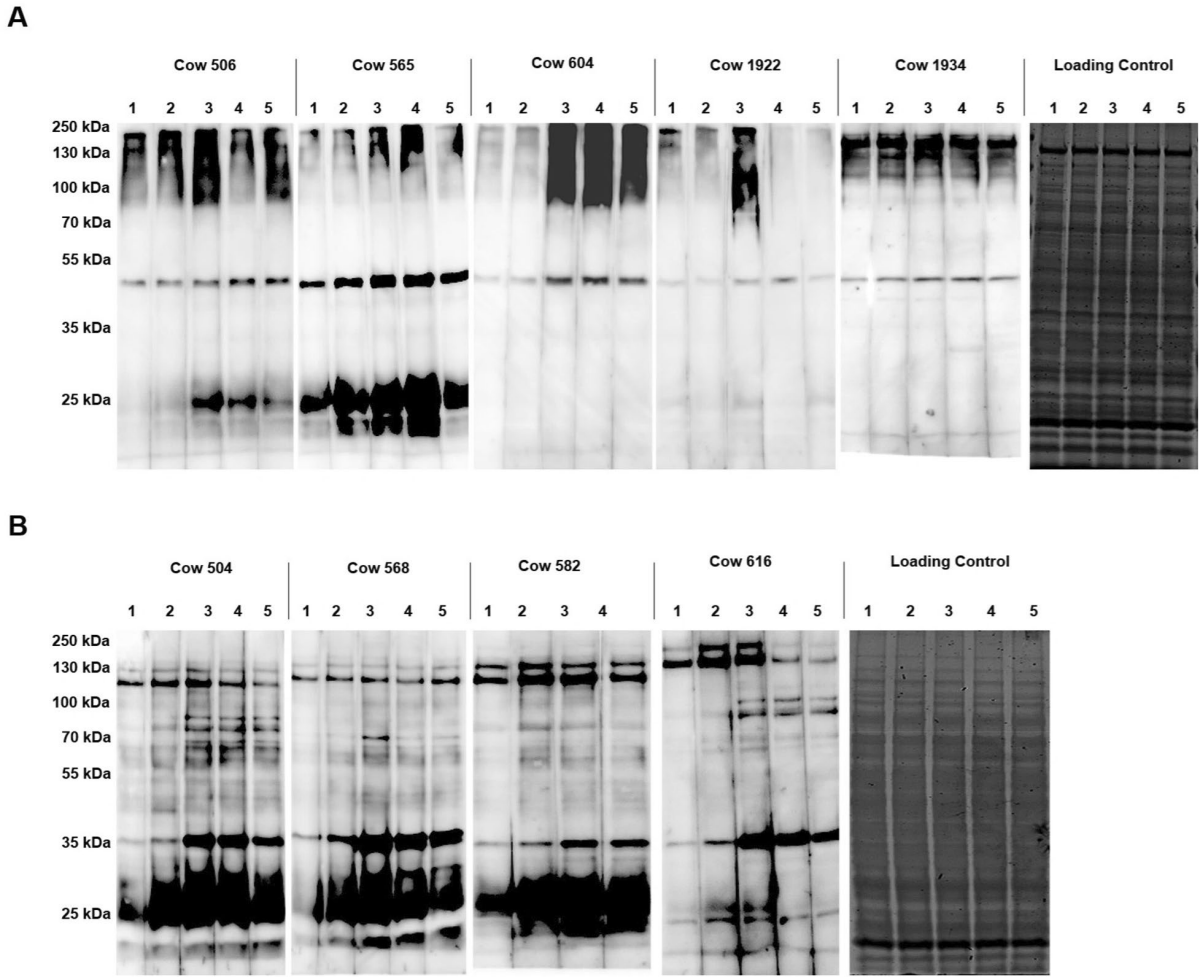
**One-dimensional serum blots of *S. aureus* cell wall-associated proteins**. **A** MOK023 cell wall-associated proteins probed with sera from MOK023 infected cows and **B** MOK124 cell wall-associated proteins probed with sera from MOK124 infected cows. Lane 1 = Day 0 pre-infection, Lane 2 = Day 7 post-infection, Lane 3 = Day 14 post-infection, Lane 4 = Day 21 post-infection and Lane 5 = Day 29 post-infection. Equal loading is shown by coomassie-stained gels of cell-wall extracts.

Cell wall protein extracts from mid-exponential phase cultures of MOK023 and MOK124 were separated by 1D SDS-PAGE and immobilised onto PVDF membrane. The proteins were probed with serum from each cow infected with that strain. Figure 1A shows the results of the MOK023 proteins probed with serum from cows infected with this strain. The humoral response predominantly targeted high molecular weight proteins, with a number of immunoreactive bands > 80 kDa. All cows in this group also had a reactive band at ~50 kDa. For two cows (506 and 565) an immune response against a number of low molecular weight proteins was also evident. Figure 1B shows the results of the MOK124 cell wall proteins probed with serum from cows infected with this strain. For this group, the induced humoral response primarily targeted low molecular weight proteins, with major immunoreactive bands identified at ~25, ~30 and ~35 kDa. (Figure 1B). A few high molecular weight weakly immunoreactive bands could also be identified (~120, ~130 and ~200 kDa); however, in many cases reactivity did not increase post-infection. Immunoreactive bands could be identified in all cows from both groups prior to intramammary challenge.

### Identification of immunogenic cell wall-associated proteins from *S. aureus* MOK023 and MOK124

The cell wall-associated proteins of MOK023 and MOK124 were separated by 2D electrophoresis and immobilised onto PVDF membrane. Proteins from both strains were probed with serum collected pre-infection and 14 days post-infection from cows 604 (MOK023 infected) and 504 (MOK124 infected) to identify both strain-specific and common antigens. Spots that increased in intensity post-infection were considered immunogenic.

**Figure 2:**
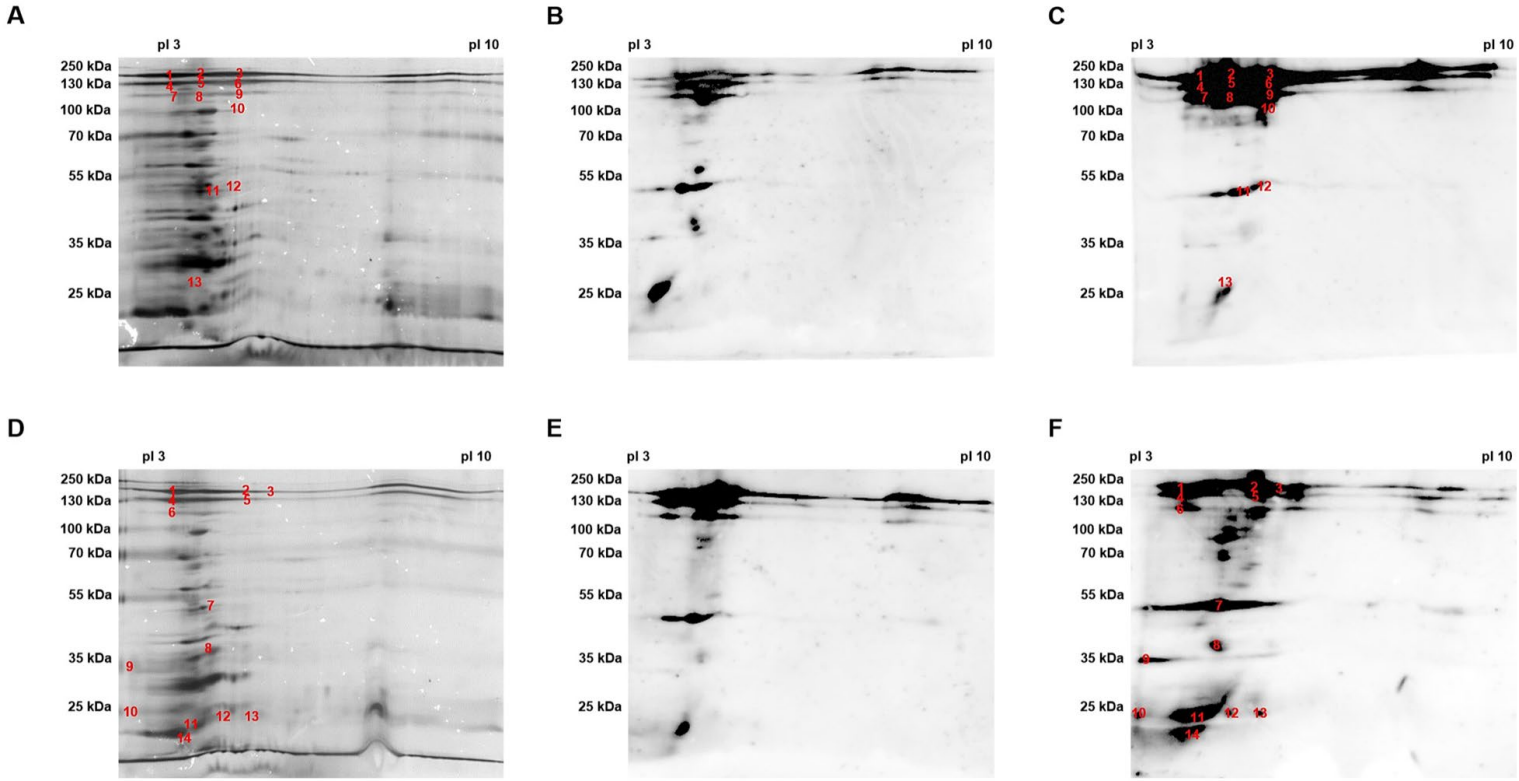
**Two-dimensional silver nitrate stained gels and serum blots of MOK023 cell wall-associated proteins**. **A** and **D** Silver nitrate stained gel of MOK023 cell wall-associated protein extract. Numbers in red indicate the spots where proteins were detected by mass spectrometry. Membranes were probed using day 0 (B & E) and day 14 **C** and **F** serum from cow 604 (MOK023 infected) **B** and **C** and cow 504 (MOK124 infected) **E** and **F**.

The results of the 2D silver nitrate stained gels and serum blots of MOK023 proteins probed with sera from cow 604 (MOK023 infected) and cow 504 (MOK124 infected) are shown in Figure 2. As seen in the 1D gels, antibodies raised in cow 604 primarily targeted high molecular weight (> 100 kDa) proteins (Figure 2C); however for cow 504, antibodies reacted against high, medium and low molecular weight proteins (Figure 2F). Antibodies raised in cow 504 also primarily targeted low p*I* proteins (Figure 2F). Candidate immunogenic antigens from MOK023 were identified by mass spectrometry analysis of spots which showed an increase in immunoreactivity post-infection. Proteins were detected in 27 excised spots, with more than one protein detected in 25 spots. However, CWA proteins could be identified in only 18 spots; the top two candidate CWA proteins identified in each spot are listed in Table 2. Additional file 2 lists all detected proteins in each of the spots.

**Table 2:** **List of MOK023 (CC97) candidate immunogenic CWA proteins detected by mass spectrometry.**

Serum source	Spot number	Protein name	# Unique peptides	Predicted localisation	Theoretical Mr (kDa)/pI *	Observed Mr (kDa)/p*I* ^+^
Cow 604 (MOK023 infected)	1	Serine-aspartate repeat-containing protein E (SdrE)	50	Cell wall	126/4.46	~180/3.5–3.9
		Fibronectin binding protein B (FnbpB)	7	Cell wall	103/4.64	
	2	Serine-aspartate repeat-containing protein E (SdrE)	52	Cell wall	126/4.46	~180/4.1–4.5
		Fibronectin binding protein B (FnbpB)	11	Cell wall	103/4.64	
	**3**	Serine-aspartate repeat-containing protein E (SdrE)	59	Cell wall	126/4.46	~180/4.8–5.0
		Serine-aspartate repeat-containing protein D (SdrD)	14	Cell wall	148/4.39	
	**4**	Serine-aspartate repeat-containing protein E (SdrE)	34	Cell wall	126/4.46	~130/3.5–3.9
		Clumping factor B (ClfB)	19	Cell wall	95/4.2	
	**5**	Serine-aspartate repeat-containing protein E (SdrE)	53	Cell wall	126/4.46	~130/4.1–4.5
		Clumping factor B (ClfB)	26	Cell wall	95/4.2	
	**6**	Serine-aspartate repeat-containing protein E (SdrE)	50	Cell wall	126/4.46	~130/4.8–5.0
		Clumping factor B (ClfB)	28	Cell wall	95/4.2	
	**7**	Serine-aspartate repeat-containing protein E (SdrE)	18	Cell wall	126/4.46	~120/3.5–3.9
		Clumping factor B (ClfB)	15	Cell wall	95/4.2	
	**8**	Serine-aspartate repeat-containing protein E (SdrE)	31	Cell wall	126/4.46	~120/4.5–4.7
		Clumping factor B (ClfB)	13	Cell wall	95/4.2	
	**9**	Serine-aspartate repeat-containing protein E (SdrE)	26	Cell wall	126/4.46	~120/4.8–5.0
		Clumping factor B (ClfB)	22	Cell wall	95/4.2	
	**10**	Serine-aspartate repeat-containing protein E (SdrE)	8	Cell wall	126/4.46	~100/4.5–5.0
		Clumping factor B (ClfB)	3	Cell wall	95/4.2	
Cow 504 (MOK124 infected)	**1**	Serine-aspartate repeat-containing protein E (SdrE)	46	Cell wall	126/4.46	~180/3.9–3.98
		Serine-aspartate repeat-containing protein D (SdrD)	8	Cell wall	148/4.39	
	**2**	Serine-aspartate repeat-containing protein E (SdrE)	46	Cell wall	126/4.46	~180/5.1–5.3
	**3**	Serine-aspartate repeat-containing protein E (SdrE)	18	Cell wall	126/4.46	~180/5.4–5.5
	**4**	Serine-aspartate repeat-containing protein E (SdrE)	47	Cell wall	126/4.46	~130/3.9–3.98
		Clumping factor B (ClfB)	23	Cell wall	95/4.2	
	**5**	Serine-aspartate repeat-containing protein E (SdrE)	21	Cell wall	126/4.46	~130/5.1–5.3
		Clumping factor B (ClfB)	11	Cell wall	95/4.2	
	**6**	Clumping factor B (ClfB)	12	Cell wall	95/4.2	~125/3.9–3.98
		Serine-aspartate repeat-containing protein E (SdrE)	8	Cell wall	126/4.46	
	**8**	Staphylococcal protein A (SpA)	3	Cell wall	41/5.53	~37/4.5–4.98
	**9**	Iron regulated surface determinant protein A (IsdA)	4	Cell wall	39/9.64	~35/3.4–3.6

^*^ Theoretical Mr/*pI* = theoretical molecular weight/isoelectric point, calculated from the amino acid sequence.

^+^ Observed Mr/*pI* = observed molecular weight/isoelectric point, based on location following 2D electrophoresis.

**Figure 3:**
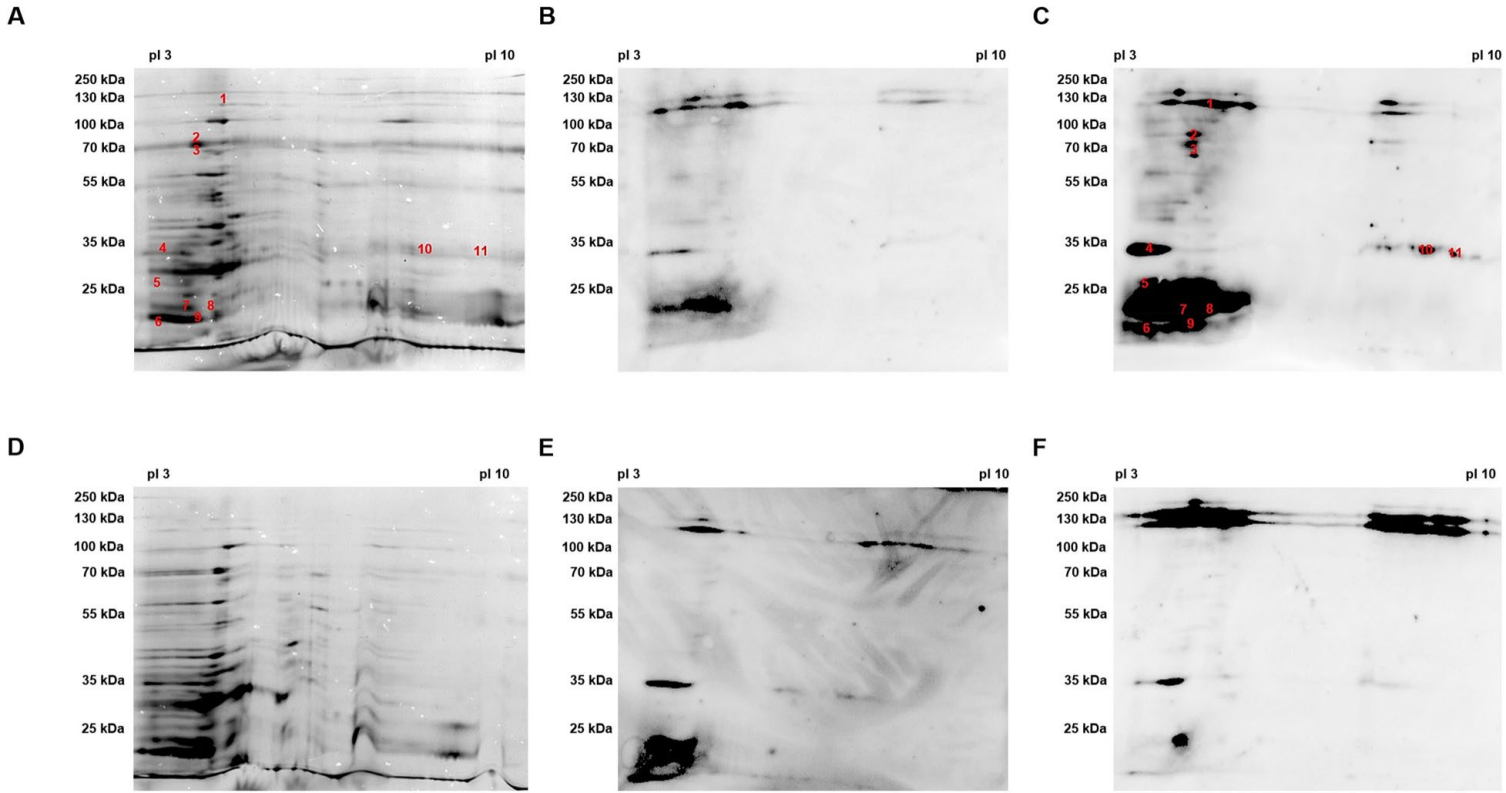
**Two-dimensional silver nitrate stained gel and serum blots of MOK124 cell wall-associated proteins**. **A** and **D** Silver nitrate stained gel of MOK124 cell wall-associated protein extract. Numbers in red indicate the spots where proteins were detected by mass spectrometry. Membranes were probed using day 0 **B** and **E** and day 14 **C** and **F** serum from cow 504 (MOK124 infected) **B** and **C** and cow 604 (MOK023 infected) **E** and **F**.

The results of the 2D silver nitrate stained gels and serum blots of the MOK124 proteins probed with sera from cow 504 (MOK124 infected) and cow 604 (MOK023 infected) are shown in Figure 3. Antibodies generated by cow 504 primarily targeted low molecular weight (< 35 kDa) proteins (Figure 3C) in agreement with the 1D analysis; however, antibodies generated by cow 604 primarily targeted high molecular weight (> 100 kDa) proteins (Figure 3F) with few common immunoreactive spots. More immunoreactivity was evident at day 14 post-infection when the MOK124 proteins were probed with serum from the cow infected with the homologous strain compared to the heterologous strain. Candidate immunogenic proteins from MOK124 were identified by mass spectrometry. Of the selected immunoreactive spots, proteins were detected in 13 of the excised spots, with more than one protein detected in 12 of those spots. However, candidate CWA proteins were detected in only 4 spots; the top candidate CWA protein identified in each spot is listed in Table 3. Additional file 3 lists all detected proteins in each of the spots. While reactive antigens were evident when MOK124 cell wall-associated proteins were probed with serum from cow 604, there was insufficient protein in many corresponding spots for candidate identification and no candidate CWA proteins were identified.

**Table 3:** **List of MOK124 (CC151) candidate immunogenic cell wall-anchored proteins detected by mass spectrometry.**

Serum source	Spot number	Protein name	# Unique peptides	Predicted localisation	Theoretical Mr (kDa)/pI *	Observed Mr (kDa)/p*I* ^+^
Cow 504 (MOK124 infected)	1	Clumping factor B (ClfB)	9	Cell wall	92/4.2	~120/4.8–5
	2	Bone sialoprotein-binding protein (Bbp)	6	Cell wall	121/4.55	~80/4.2–4.5
	10	Iron regulated surface determinant protein A (IsdA)	2	Cell wall	39/9.6	~35/8.1–8.6
	11	Iron regulated surface determinant protein A (IsdA)	2	Cell wall	39/9.6	~35/10.1–10.3

^*^ Theoretical Mr/*pI* = theoretical molecular weight/isoelectric point, calculated from the amino acid sequence.

+ Observed Mr/*pI* = observed molecular weight/isoelectric point, based on location following 2D electrophoresis.

### Immunoreactivity of candidate antigens

**Figure 4:**
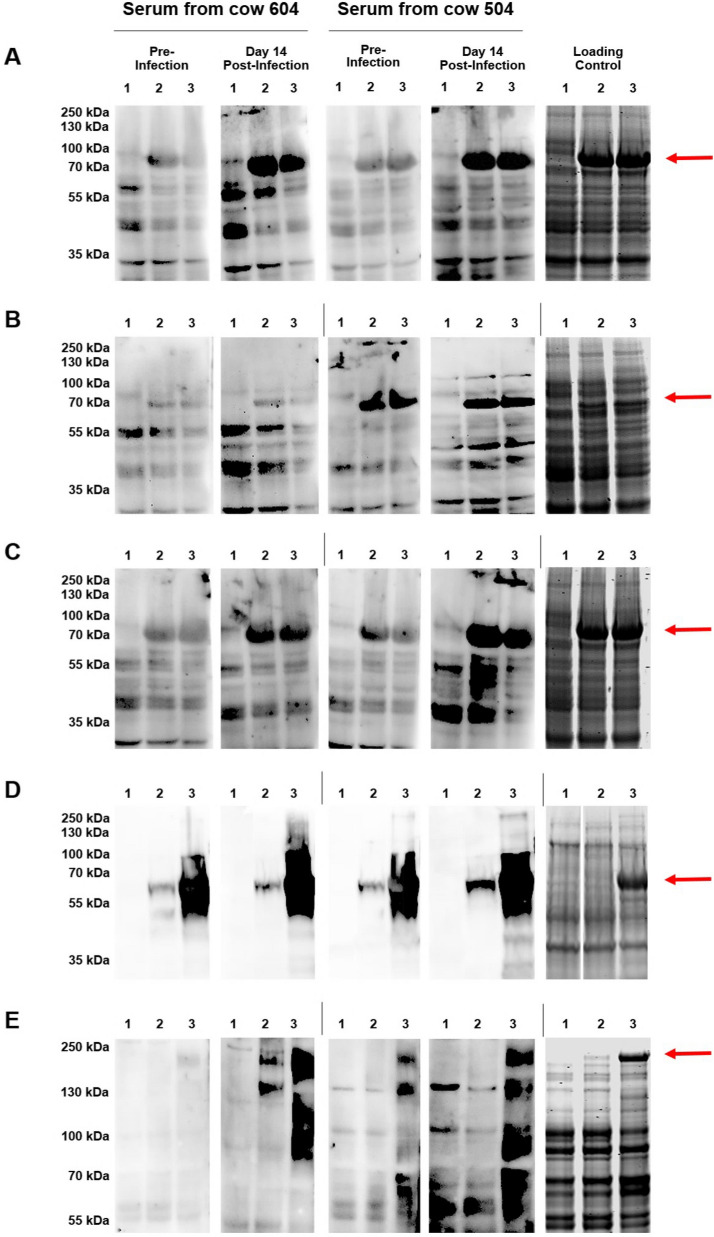
**One-dimensional serum blots of lysates of *E. coli* BL21 overexpressing recombinant proteins**. Lysates of *E. coli* BL21 overexpressing recombinant ClfA (**A**), ClfB (**B**), FnbpB (**C**), IsdA (**D**) and SdrD (**E**) were probed using sera from cow 604 (MOK023 infected) and cow 504 (MOK124 infected). Lane 1 = No plasmid control, Lane 2 = Uninduced control and Lane 3 = Induced with 100 μM IPTG for 3 h. Equal loading is shown by coomassie-stained gels of the whole cell lysates.

The 2D serum blotting identified a number of candidate immunogenic *S. aureus* CWA proteins. However, peptides matching up to 4 different CWA proteins were found in some spots and identification of specific candidates was complicated by the fact that many CWA proteins have similar molecular weight and p*I* and include repeat regions. Therefore, validation of candidate CWA proteins was performed. Whole cell lysates of *E. coli* expressing recombinant ClfA, ClfB, FnbpB, IsdA or SdrD were separated by 1D electrophoresis, immobilised onto PVDF membrane and probed with serum collected pre and post-infection from cows 604 and 504. The results of the serum blots of lysates of untransformed *E. coli* as well as *E. coli* expressing recombinant ClfA, are shown in Figure 4A and Additional file 4. ClfA was highly expressed, even in the absence of induction. Sera from both cows demonstrated minimal reactivity with rClfA pre-infection; however, a strong anti-ClfA response was induced post-infection in both cows. Cow 604 induced a weak antibody response to ClfB post-infection that was most evident 21 days post-infection (Figure 4B and Additional file 5). In contrast, cow 504 had antibodies to ClfB pre-challenge indicating prior exposure to ClfB-expressing *S. aureus*; reactivity increased post-infection, most notably at day 29 (Additional file 5). Both cows had minimal immunoreactivity against FnbpB pre-infection but induced a strong antibody response post-infection (Figure 4C and Additional file 6). For IsdA, strong immunoreactivity was evident for both cows pre-infection and reactivity increased post-infection (Figure 4D and Additional file 7). Both cows also generated anti-SdrD antibodies post-infection, with cow 504 showing evidence of prior exposure to SdrD (Figure 4E and Additional file 8).

## Discussion

In this study, a proteomic approach was taken to characterise the immunogenicity of CWA proteins of *S. aureus* strains MOK023 (CC97) and MOK124 (CC151). Genome sequence analysis demonstrated that MOK023 encoded more CWA proteins than MOK124 due to extensive decay in genes encoding CWA proteins in MOK124. This is in agreement with previous studies of strains belonging to CC151 [6, 7, 36]. Allelic variation in a number of genes encoding CWA proteins was also identified. Such variation may affect their antigenicity, as has been shown for FnbpA and FnbpB [37, 38]. The humoral immune response to cell wall proteins of these contrasting strains was characterised using pre and post-infection serum from a bovine intramammary challenge trial [16]. The results from 1D serum blots showed that the strains induced, to some extent, a strain-specific response. Cows infected with MOK023 produced antibodies that mainly reacted against high molecular weight proteins while cows infected with MOK124 produced antibodies that mainly reacted against low molecular weight proteins. The serum blots also indicated the cows had prior exposure to *S*. *aureus*; however, this was expected as the cows used in this study were colonised by *S. aureus* at extramammary sites at the time of infection (unpublished observation). Despite this, there was a distinct increase in serum reactivity against a number of CWA proteins after intramammary challenge. Interestingly, it was also noted in the serum blots that there was a decline in immunoreactivity to some proteins on or before day 29 post-infection, despite the fact that the cows were still infected. *S*. *aureus* is known to cause chronic, persistent infections [39, 40]. Strategies used by *S*. *aureus* for evasion of the host immune system include internalisation within host cells and biofilm formation [30, 41–44]. Thereby, *S*. *aureus* can evade intracellular killing by the phagosome, effector molecules and cells of the immune system as well as avoid killing by antibiotics. While *S*. *aureus* are internalised or biofilm-associated, limited antigens may be available for recognition by the immune system and this could be responsible for the reduced serum reactivity. Alternatively, within-host adaptation may have resulted in a change in the phenotypic traits expressed in vivo over the course of such a chronic, persistent infection [45].

In order to identify specific immunogenic proteins expressed in vivo by MOK124 and MOK023, 2D electrophoresis followed by serum blotting was used for cell wall protein separation and protein identification. Serum from cows 504 and 604 only were used to probe the cell wall-associated proteins as these 2 cows typified the IgG response within each group. Serum from a single cow from each group was used for the immunoblot analysis rather than a pooled serum sample as the genotype of *S. aureus* to which each cow had been previously exposed was unknown. Serum from a single cow was therefore considered preferable to identify spots that increased in intensity post-infection. However, the use of serum from a single individual may have resulted in the identification of cow-specific antigens compared to pooled serum, which may have identified a greater diversity of antigens more representative of the response in all cows. Agreement between 1 and 2D serum blotting techniques was seen. In both instances serum from cow 504 reacted with many low molecular weight proteins while serum from cow 604 reacted predominantly with high molecular weight proteins. Notably, antibodies produced by cow 604 could cross react with cell wall-associated proteins produced by MOK124 and vice versa, indicating some common immunoreactive proteins. Common immunogenic CWA proteins identified included ClfB, SdrE/Bbp and IsdA. In addition ClfA, FnbpB and SdrD were identified when MOK023 proteins were probed. Some considerations should be taken into account when interpreting these results. Firstly, CWA proteins were harvested from early to mid exponential phase cultures grown in tryptic soy broth with aeration. These growth conditions were selected as they are conducive to the in vitro expression of a number of important CWA proteins, including the FnBPs and ClfB [46, 47]. However, they do not mimic in vivo growth conditions in the mammary gland. The repertoire of *S. aureus* CWA proteins expressed in vivo during IMI may differ from those expressed, and hence available for detection, in the present study. Growth of the *S. aureus* strains in milk or under conditions more similar to those in the mammary gland, such as iron and oxygen restriction, may have revealed additional immunogenic antigens [48]. Secondly, low intensity spots were not excised for mass spectrometry analysis due to the likelihood they would contain insufficient protein for identification. Silver nitrate staining is a sensitive method for protein detection; however, it limits protein identification to spots with medium to high intensity staining. For example, no immunogenic proteins were detected when MOK124 cell wall-associated proteins were probed with serum from cow 604. An alternative, sensitive staining method, with better compatibility with mass spectrometry, may have allowed identification of additional proteins. Thirdly, more than one protein was detected by mass spectromtry in the majority of excised spots. CWA proteins were selected as the most likely immunogenic proteins due to their cell surface expression. However, CWA proteins were not the only type of proteins detected, with many cytoplasmic membrane and cytosolic proteins also identified. This is a common finding; other studies that used enzymatic-dependent cell surface shaving methods also reported cytoplasmic contamination, which may be at least partly attributable to autolysis of *S. aureus* cells and the subsequent reattachment of cytoplasmic proteins to the cell surface [49–52]. However, serum blotting of *E. coli* lysates overexpressing SirA, a cytoplasmic membrane protein commonly found in immunoreactive spots with IsdA, found no immunoreactivity to this protein (data not shown) providing some support for the selection of CWA proteins as candidate immunogens. Future work, including the construction of isogenic *S. aureus* deletion mutants for each of the candidate immunogens, would facilitate confirmation of the identity of the immunoreactive protein in each of the identified spots.

In order to validate a number of the candidate immunogens, plasmids expressing the CWA proteins ClfA, ClfB, FnbpB, SdrD and IsdA were transformed into *E. coli* for recombinant protein expression. Lysates containing the recombinant proteins were probed with sera from cows 504 and 604. Both cows had antibodies that reacted against all recombinant proteins. Serum from cow 504 could react with SdrD and FnbpB which was unexpected as MOK124 does not encode *sdrD* or *fnbB*. One likely explanation is that antibodies raised against Bbp can cross react with SdrD; these allelic variants are 87.4% identical between the two strains. Similarly, MOK124 encodes *fnbA*, and antibodies raised against the protein likely cross-reacted with the closely related FnbpB [53]. Non-specific interactions were also seen with some *E. coli* proteins, which has been reported previously [54, 55]. Purification of the recombinant proteins prior to serum blotting would likely have eliminated the detection of such bands, while also enabling downstream functional characterisation of the candidate immunogens as well as investigation of their interactions. It was notable that expression of some recombinant proteins was seen in the absence of inducer, in particular proteins expressed from pQE30. This was likely caused by promoter leakage due to insufficent copies of the *lacI* repressor, which is not encoded by pQE30 and is not highly expressed by *E. coli* BL21 DE3 cells [56].

Infection with MOK023 or MOK124 has been demonstrated to result in contrasting host response and infection signs [16]. The infecting *S. aureus* strain has also been demonstrated to influence the severity and outcome of bovine IMI in a variety of other studies [40, 45, 57]. These studies suggest that particular strains or genotypes express distinctive phenotypic traits during the course of infection. Therefore, different strains may have different mechanisms by which they infect or colonise the mammary gland and cause disease. In a previous study we demonstrated that MOK124 produced more toxins in vitro than MOK023 and the secreted proteins from MOK124 against which a serum IgG response was targeted during bovine IMI included a number of toxins [28]. The results from this study suggest that the serum IgG response to MOK023 targets many high molecular weight proteins, most likely CWA proteins, further supporting the hypothesis that these strains have contrasting modes of pathogenicity.

A number of the immunogens identified in this study have been previously tested as vaccine candidates designed to prevent *S. aureus* IMI. The ability of recombinant ClfA + IsdA and ClfA + FnBPA to induce a serum and milk IgG response in vivo has been demonstrated and the functional capacity of the antibodies demonstrated [20, 58]. Vaccine efficacy trials have also indicated the ability of these proteins to provide at least partial protection against *S. aureus* IMI [21, 59]. The results from the present study indicate that strains from both major bovine-adapted lineages CC97 and CC151 produce these immunogenic antigens in vivo, supporting their investigation as pan-lineage protective antigens. A number of methods, such as peptide arrays or yeast, bacterial or phage display systems, are currently available to determine the epitopes recognised by antibodies. Future work should include the application of such technologies for the identification of immunodominant regions of the candidate CWA proteins, as well as the identification of novel immunogens. However, the extensive sequence variation between strains and lineages will need to be considered with this approach.

The development of novel mastitis management and control strategies requires knowledge of the prevalent pathogens and an understanding of the mechanism by which they cause disease. New strategies must target all major strains or genotypes to maintain cow health and welfare and milk quality as dairy producers rarely have information on the genotype of the infecting strain. Therefore, this study, which may have implications for the development of vaccines against different *S. aureus* strains and lineages, offers a starting point for further research into the mode of pathogenicity of prevalent bovine-adapted strains as well as the utility of CWA proteins as vaccine or diagnostic candidates. However, some considerations should be made for future work. Quantitative analysis could help to determine if commonly identified proteins between different *S. aureus* strains share similar immunoreactivity or not. In addition, the ability of the antibodies generated to provide protection from infection must be further explored. Moreover, antibodies produced in response to one strain may cross-react with, but not cross-protect against, other *S. aureus* strains and this remains to be determined. The role of other antibody classes, particularly locally produced IgA and IgM should be considered as important mammary gland defense and natural antibody classes [60]. Furthermore, additional CC97 and CC151 *S. aureus* strains, along with strains from other important bovine-adapted lineages, should be investigated to identify common and strain-specific immunogenic proteins.

### Supplementary Information

**Additional file 1: Repertoire of known cell wall anchored proteins in the genome of MOK023 and MOK124.**

**Additional file 2: Mass spectrometry data for all MOK023 proteins identified in spots excised from 2D gels of MOK023 surface proteins. **Equivalent blots were probed with serum from cow 604 (MOK023 infected) and cow 504 (MOK124 infected) to identify immunogenic proteins.

**Additional file 3: Mass spectrometry data for all MOK124 proteins identified in spots excised from 2D gels of MOK124 surface proteins. **Equivalent blots were probed with serum from cow 504 (MOK124 infected) and cow 604 (MOK023 infected) to identify immunogenic proteins.

**Additional file 4: One-dimensional serum blots of lysates of E. coli BL21 (DE3) overexpressing recombinant ClfA. **Membranes were probed using sera from cow 604 (MOK023 infected) and cow 504 (MOK124 infected).

**Additional file 5: One-dimensional serum blots of lysates of E. coli BL21 (DE3) overexpressing recombinant ClfB. **Membranes were probed using sera from cow 604 (MOK023 infected) and cow 504 (MOK124 infected).

**Additional file 6: One-dimensional serum blots of lysates of E. coli BL21 (DE3) overexpressing recombinant FnbpB. **Membranes were probed using sera from cow 604 (MOK023 infected) and cow 504 (MOK124 infected).

**Additional file 7: One-dimensional serum blots of lysates of E. coli BL21 (DE3) overexpressing recombinant IsdA. **Membranes were probed using sera from cow 604 (MOK023 infected) and cow 504 (MOK124 infected).

**Additional file 8: One-dimensional serum blots of lysates of E. coli BL21 (DE3) overexpressing recombinant SdrD. **Membranes were probed using sera from cow 604 (MOK023 infected) and cow 504 (MOK124 infected).

## Data Availability

All data generated or analysed during this study are included in this published article and its additional information files.
