# Supplementary material for: Immunoproteomic analysis of the serum IgG response to cell wall-associated proteins of Staphylococcus aureus strains belonging to CC97 and CC151
Source: Vet Res. 2023 Sep 18;54:79. doi: 10.1186/s13567-023-01212-7 (PMC10506246; doi:10.1186/s13567-023-01212-7)
Supplement: Supplementary file 8 — Additional file 8: One-dimensional serum blots of lysates of E. coli BL21 (DE3) overexpressing recombinant SdrD. Membranes were probed using sera from cow 604 (MOK023 infected) and cow 504 (MOK124 infected). [file 13567_2023_1212_MOESM8_ESM.pdf]

A

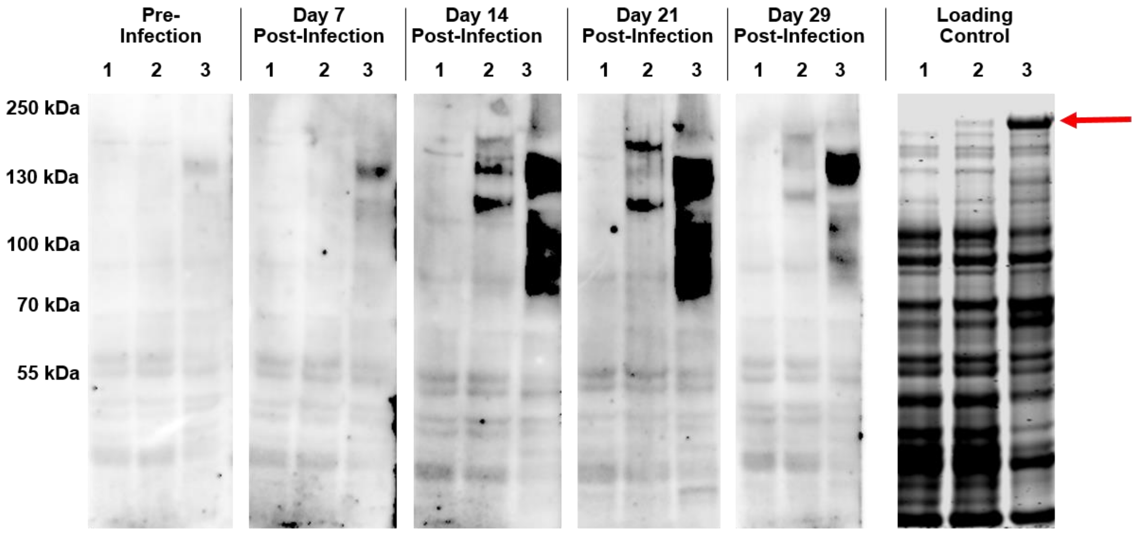

B

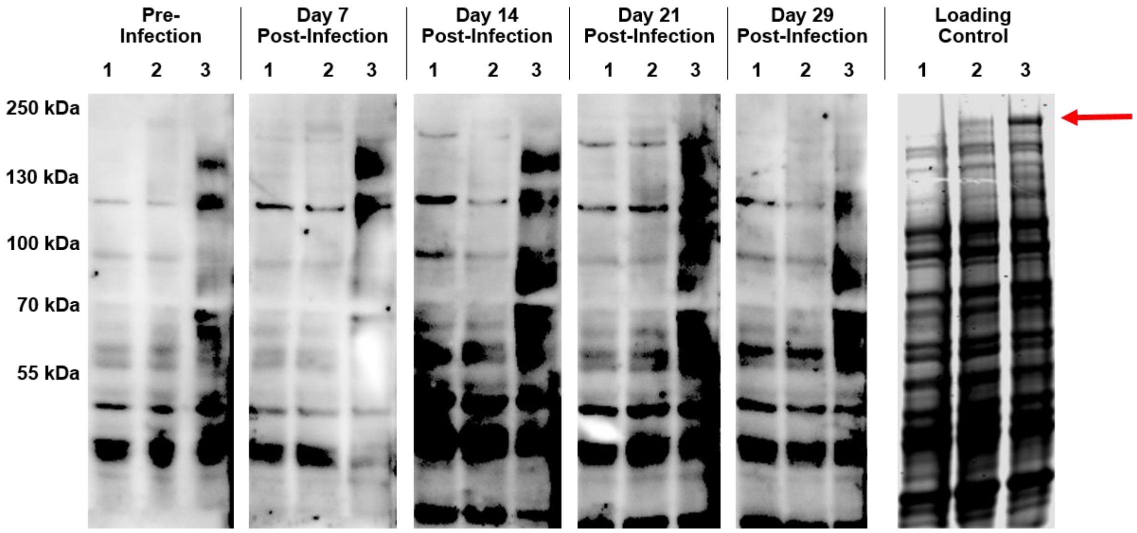

Additional file 8: One-dimensional serum blots of *E. coli* BL21 (DE3) overexpressing recombinant SdrD.

Membranes were probed using sera from **(A)** cow 604 (MOK023 infected) and **(B)** cow 504 (MOK124 infected). Lane 1 = No plasmid control, Lane 2 = Uninduced control and Lane 3 = Induced with 100 µM IPTG for 3 h.
